# Photoinduced Local Symmetry Breakage in SrTiO_3_ and Potential Pathways to Ferroelectricity

**DOI:** 10.1021/acs.jpcc.4c06242

**Published:** 2025-01-23

**Authors:** Elena R. Remesal, Víctor Posligua, Jose J. Plata, Antonio M. Márquez

**Affiliations:** Departamento de Química Física, Facultad de Química, 16778 Universidad de Sevilla, 41012 Seville, Spain

## Abstract

During the past few years Strontium Titanate, STO, has been the subject of extensive studies exploring the phase transitions it undergoes from paraelectric to ferroelectric or antiferrodistortive phases, induced by electromagnetic radiation such as lasers. This ability to exhibit photoinduced ferroelectricity has profound implications for its potential applications in photovoltaic and optoelectronic devices. Additionally, SrTiO_3_ exhibits a rich dielectric behavior under UV irradiation in the presence of an electric field. In this work, we explore the nature of the photoinduced enhancement of the STO dielectric constant under UV irradiation. Analyzing the STO potential energy surface (PES), the first excited states exhibit a local breaking of symmetry due to the displacement of the Ti atom off the center of the unit cell. Furthermore, two key variables linked to the ferroelectric phase transition in STO, namely, pressure and oxygen vacancies, are investigated. Pressure can modify the ground state and excited state PES, tailoring the depth of the excited state well or reducing the energy gap between the ground and excited states. The distribution of oxygen vacancies in the STO bulk was also explored, revealing preferential configurations and the presence of aligned Ti^3+^-O_vac_-Ti^3+^ or Ti^3+^-O_vac_ complexes. These configurations can lead to a small polarization of the material, which could serve as a driving force to align the off-center photoexcited Ti polarons.

## Graphical abstract

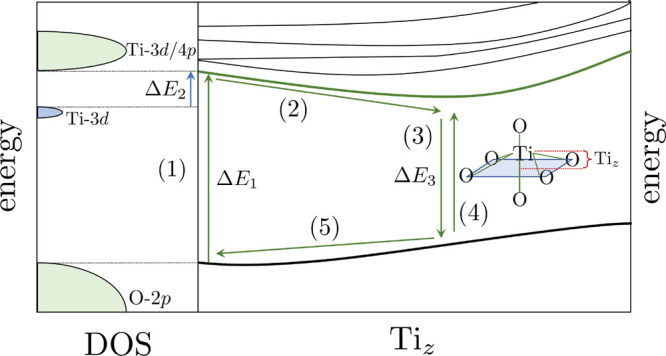

## Introduction

From the late 1970s to the early 2000s, the efficiency of traditional photovoltaic (PV) cells based on single crystal Si, GaAs, or CdTe has evolved from approximately 8–13 to 25%.^1^ Since then, improvement in the efficiency of these classic solar cells based on p–n junctions seems to have reached a plateau. The discouraging progress during the past decade stems from the limited photovoltage of solar cells based on p–n junctions, which is due to the band gap of the semiconductor and the Shockley–Queisser, S-L, limit.^2^ To make this technology more competitive, significant improvements in efficiency must be achieved through the discovery of new PV mechanisms that overcome these limitations. Ferroelectric materials, which exhibit spontaneous electric polarization, demonstrate different behavior under photoexcitation, providing an alternative approach to separate charge carriers.^3^ This phenomenon is known as the bulk PV effect,^4,5^ which has reinvigorated efforts for the development of new solar cells based on ferroelectric materials.^6^

A wide range of ferroelectric materials have been explored for their potential applications in solar cell devices. Hybrid perovskite thin films such as CH_3_NH_3_PbI_3_ ^7–10^ have revolutionized the solar cell field in recent years due to their low cost and high efficiency.^11–13^ However, their low stability and durability have hindered their implementation in the market. Chalcohalides have been recently proposed as an alternative,^14^ but they typically exhibit lower polarization than other ferroelectric materials.^6^ Oxide perovskites stand out as the most promising candidates, as their strong polarization and high thermal, mechanical, and chemical stability are combined with very low synthesis costs.^15,16^ Nevertheless, oxide perovskites also present challenges that must be overcome to design efficient PV devices, with their wide band gap being the primary obstacle.^17^

SrTiO_3_, STO, is one of the most extensively studied oxide perovskites.^18–20^ Its structure and optoelectronic properties have been thoroughly examined through experimental and theoretical studies.^18,21,22^ Consequently, the methods to tailor its band structure and band gap are well-known.^23–25^ Although SrTiO_3_ is inherently paraelectric and centrosymmetric, even at very low temperatures, various strategies such as doping,^26^ isotope substitution,^27^ or Sr/Ti nonstoichiometry,^28^ have been shown to be effective in achieving a ferroelectric phase transition. Nonetheless, strain appears to be the most straightforward approach to observe ferroelectric behavior in STO at room temperature.^29,30^ Recently, Alexe et al. reported the observation of the bulk PV effect in STO when strain gradients are introduced using an atomic force microscope tip.^31^ As an alternative to strain, optical excitation can also induce ferroelectricity in STO.^32^ Li et al. demonstrated that intense terahertz electric field excitation can induce a ferroelectric phase in STO.^33^ Furthermore, Nova et al. reported the observation of a ferroelectric metastable phase in STO that persists for hours after mid-infrared optical pulses were used to excite lattice vibrations, with this ferroelectric state stable up to 290 K.^34^ To gain additional insight into these intriguing photoinduced effects, different theoretical works have been published in recent years.^35^ For instance, Linker et al. performed nonadiabatic quantum molecular dynamics that revealed picosecond amorphization in photoexcited STO.^36^ Additionally, state-of-the-art first-principles were used by Latini et al. to study optical cavities in STO as responsible for the strong interaction between light and matter.^37^

In addition to the ferroelectric phase, STO presents rich dielectric behavior. When STO is cooled below 100 K, a rise in its dielectric function indicates an antiferrodistortive (AFD) second-order structural phase transition.^38^ The oxygen octahedra of adjacent unit cells within the basal plane exhibit an antiphase rotation around the Ti atom, resulting in a tetragonal distortion.^39,40^ The competition between AFD and ferroelectric instabilities in STO has been well established.^41,42^ The distortion of the oxygen octahedra leads to antipolar displacements of the Sr site, which in turn suppresses the ferroelectric instability in this material.^43^ AFD transitions can also be laser-induced through optical pulses in the order of a few picoseconds.^44^ At low temperatures, STO also exhibits a significantly enhanced dielectric constant under ultraviolet irradiation in the presence of an electric field.^45,46^ This behavior is not attributed to an AFD state, but rather to local symmetry breaking,^47–49^ where the dielectric dipole moment arises from the off-center displacement of the Ti atoms.^50^

While most theoretical studies focus on the STO ferroelectric transition induced by resonant terahertz laser pulses or the competition between AFD and ferroelectric states, to our knowledge, there are no theoretical models that explore the local symmetry breaking in STO arising from the combination of UV excitation and an applied electric field. In this work, a cluster model and time-dependent density functional theory (DFT) calculations are combined to understand this phenomenon. Additionally, different strategies are explored to potentially break the spatial symmetry and create a ferroelectric phase in the absence of an external electric field.

## Methodology

### Periodic DFT Calculations

SrTiO_3_ lattice was modeled using periodic DFT calculations within the VASP package^51–54^ and the projector augmented wave (PAW) method.^54,55^ Energies were calculated with the generalized gradient approximation (GGA) proposed by Perdew et al.^56^ and a plane-wave basis set with a 500 eV cutoff. DFT+U formalism was used to represent the electronic structure of the 3*d* states of Ti atoms. The Hubbard *U* term was included using the rotationally invariant approach proposed by Dudarev et al.^57^ in which the Coulomb *U* and the exchange *J* parameters are combined into a single parameter *U*_eff_ = *U*–*J*. A *U*_eff_ parameter of 4.5 eV was selected for the Ti-3*d* states, following previous studies on SrTiO_3_ ^58^ and standards proposed by Calderon et al.^59^ The Brillouin zone of the primitive cell was sampled using a 10 × 10 × 10 Γ-centered mesh. The Hellmann–Feynman theorem was used to calculate the forces over the ions, including the Harris-Foulkes corrections.^60^ The initial crystal structure was considered fully relaxed (cell volume and atom coordinates) when the forces over all atoms were less than 10^–6^ eV Å^–1^, obtaining a lattice parameter, *a*, of 3.9695 Å. This value is closely in agreement with the experimental value, 3.898 Å, at low temperatures.^61^

### Cluster Model

Using the bulk structures obtained in the aforementioned DFT+U calculations, a cluster model was built in the following way. First, a Sr_8_Ti_7_O_8_ cluster of atoms centered on a Ti was extracted from the bulk and surrounded by an array of 3454 point charges (PCs) of nominal values for Ti and Sr (i.e., +4 for Ti and +2 for Sr) and adjusted to −1.99238 for the O atoms to preserve electroneutrality. This array of PCs is required to include the effect of the Madelung potential of the extended SrTiO_3_ solid, which is known to be largely ionic. The Sr and Ti atoms at the frontier of this cluster do not have basis functions associated with them but are represented by a large core effective core potential (ECP) that provides a simple representation of the ionic core.^62,63^ These ECPs include a short-range repulsive potential that interacts with the electrons in the internal region, avoiding an excessive polarization of the electronic cloud toward the PCs near the interface. The wave function of the central Ti and O atoms in the cluster is described by Gaussian-type basis sets. The central Ti atom is treated with the LANL2DZ basis and ECP.^64^ This ECP explicitly includes 3*s* ^2^ 3*p* ^6^ 3*d* ^2^ 4*s* ^2^ electrons in the valence shell. The basis set is of double-ζ quality, and a diffuse *s* function (ζ = 0.010467) and *p* function (ζ = 0.005333) were included. In particular, the diffuse *p* function was found to be essential to describe the Ti-4*p* levels. The O atoms are treated all-electron with a standard 6-31+g­(d) basis set. Time-dependent DFT calculations were performed with the Gaussian 09 program^65^ to obtain up to 100 excitation energies of states. The Heyd–Scuseria–Ernzerhof functional, HSE06,^66,67^ was used in all cluster calculations.

## Results and Discussion

### Potential Energy Surfaces (PESs)

Figure 1a presents the PESs obtained for the first 100 electronic states of SrTiO_3_ as a function of the displacement of the central Ti atom relative to the plane of the four equatorial O atoms, which we define as the *xy* plane (Figure 1b). For these PES, only the Ti *z* coordinate is modified, while all other atoms remain in their ground state (GS) equilibrium positions. The GS energy is plotted in black, and as expected, it increases smoothly with the displacement of the Ti atom. The energy difference between the GS and the first excited state (3.28 eV) is in excellent agreement with the indirect experimental STO band gap (3.25 eV).^21^

**Figure 1:**
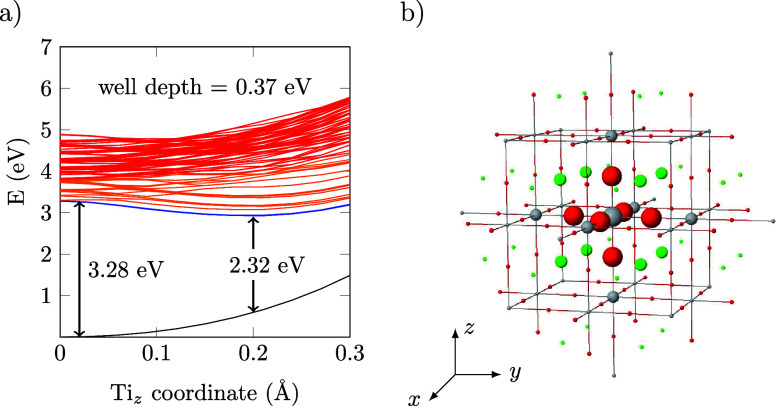
(a) Potential energy surfaces (PESs) for the SrTiO_3_ cluster model. Electronic states; Black: ground state. Orange/Blue: Ti-4*p* states. Red: Ti-3*d* states. (b) Scheme of the cluster model. Color codes: gray, Ti; red, O; and green, Sr. Larger spheres represent cluster atoms; medium-sized spheres represent ECP; and small dots represent point charges.

There is a group of four nearly degenerate states, represented in blue, whose energy shows a minimum at *z*_Ti_ ≃ 0.2 Å and a well depth of ∼0.37 eV. The energy gap between the first excited states and the GS at the deepest point of the well is below 2.4 eV, which represents a significant reduction compared to the band gap for centrosymmetric STO (3.28 eV). These states correspond to transitions from the highest energy-occupied molecular orbitals (at the top of the valence band), which are primarily the O-2*p* states, to empty Ti-4*p*_*x*,*y*_ levels. The well for these first excited states when the Ti atom is displaced along the can be explained by the molecular orbitals involved in the excitation. The electronic excitation builds up electron density on the 4*p*_*x*,*y*_ orbitals that point toward the electronic clouds of the equatorial O atoms, creating an electrostatic repulsion that can be reduced by the displacement of the Ti atom out of the equatorial O plane. The role of Ti-4*p* orbitals in the photoexcitation of STO has been previously reported by Nozawa et al. using X-ray emission spectroscopy.^49^ Slightly higher in energy, there is a set of about 23 states, pictured in orange (Figure 1a), that can be described as electronic transitions from the O-2*p* levels (valence band) to the Ti-4*p* levels (lower states of the conduction band). The next group of states at higher energy is depicted in red (Figure 1a) and corresponds to transitions from the O-2*p* levels to the empty Ti-3*d* states.

Our cluster model is able to explain the symmetry-breaking distortion of SrTiO_3_ induced by UV photoexcitation, which is in agreement with experimentally reported data. The large enhancement of the dielectric constant is observed only in the presence of a weak electric field when the displacement of the Ti atoms out of their centrosymmetric position is coordinated by the direction of the electric field. Applying an electric field breaks the cubic symmetry of the system, establishing a preferential direction for macroscopic polarization. In the following sections, we explore alternatives to applying an electric field in order to facilitate the emergence of a ferroelectric phase under UV irradiation. In the following sections, we will explore alternative approaches to inducing ferroelectricity in SrTiO_3_ under UV irradiation, focusing on the use of pressure and stoichiometry modifications, which have already been reported as effective strategies.

### Strain Effects

Epitaxial strains or other sources of strain have provided pathways to tune the electronic properties of STO.^68^ Ferroelectricity in STO is also linked to strain,^29,69^ that is why, the effect of strain in the PES of photoexcited STO has also been analyzed. Three different strains were applied: volumetric, equatorial, and axial. Volumetric strain corresponds to an isotropic deformation of the unit cell, while equatorial and axial strain represent deformations perpendicular or parallel to Ti displacement, respectively. PESs were calculated for these three kinds of distortions up to 3% of compression (negative strain) and tension (positive strain) of their lattice constants. The most important features of the PES under strain (centrosymmetric band gap, noncentrosymmetric band gap, well position, and well depth) are included in Figure 2. It is important to notice that centrosymmetric bandgaps cannot be directly compared with previous periodic calculations^70^ because the positions of atoms are not relaxed in our embedded cluster model. The main purpose of this section is not to reproduce previous works but to understand the modification of band gaps of STO considering the displacement of the Ti atom when the system has been photoexcited.

**Figure 2:**
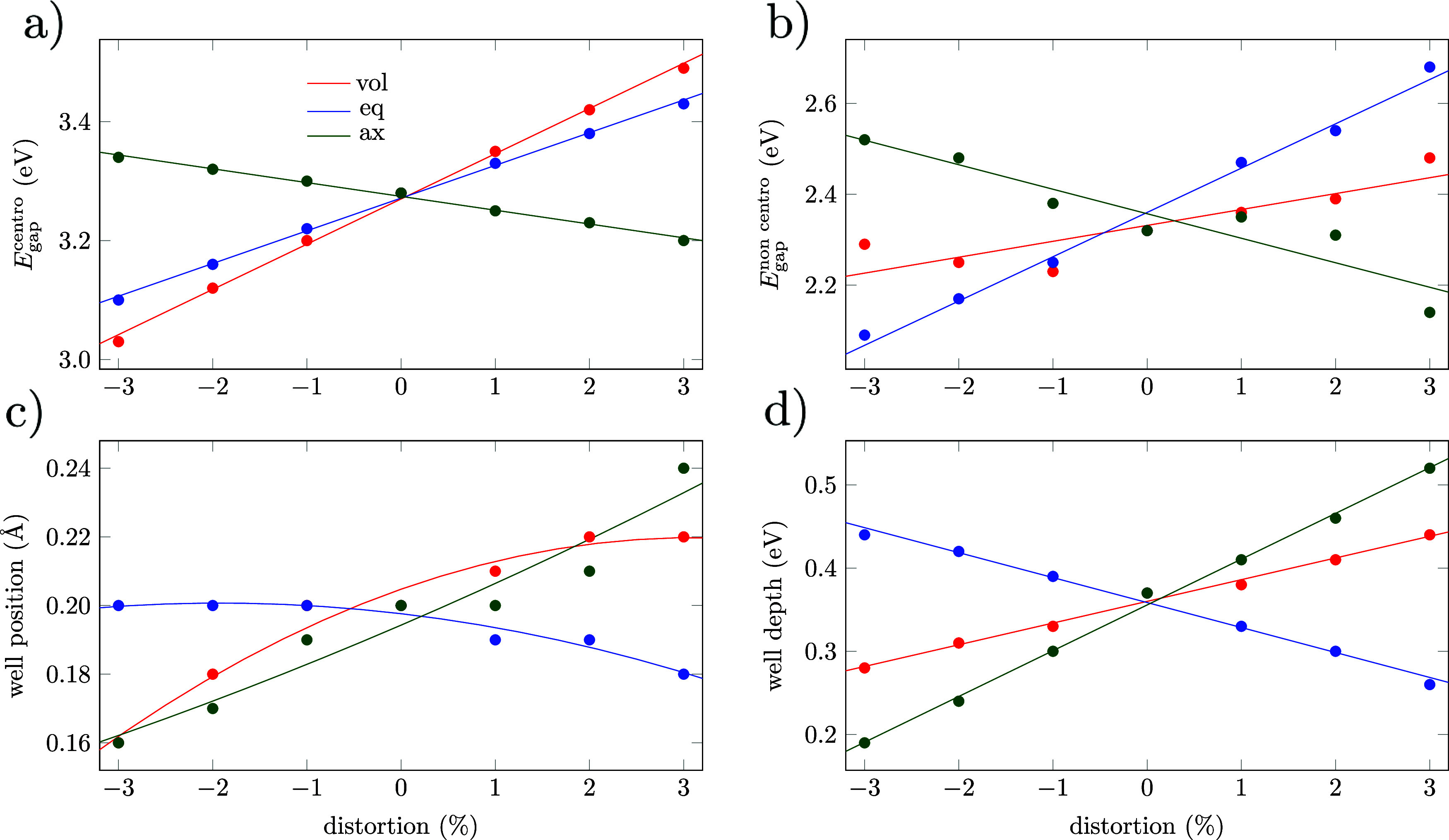
(a) Band gap energy (eV) for centrosymmetric STO under different strains. (b) Band gap energy (eV) for noncentrosymmetric STO where the Ti-*z* coordinate is determined by the lowest energy of the first excited state. (c) Ti-*z* (Å) value for the lowest energy of the first excited state (well position). (d) Well depth (eV) of the first excited state. Colors: red = isotropic volumetric strain; blue, equatorial strain; and green, axial strain.

An important reduction of the energy gap is predicted for any kind of strain upon comparison of Figure 2a,b. The reduction of the gap between the centrosymmetric and noncentrosymmetric positions depends on three main features: the shape of the GS PES (see Figure 1a) the position and the depth of the well of the first excited state (see Figure 2c,d, respectively). The displacement of the well position can be rationalized by taking into account that although the displacement of the Ti atom off the equatorial plane reduces the repulsion between the electron located at the Ti-4*p*_*x*,*y*_ orbital in the excited state, at some point the repulsion with the apical O atom stops this displacement. When strain is applied equatorially, a negative strain increases the repulsion between the Ti-4*p*_*x*,*y*_ electron and the equatorial O atoms. However, since the apical O atom is not displaced in our model, the maximum Ti atom displacement remains similar. When the strain is positive, the repulsion between the Ti-4*p*_*x*,*y*_ electron and the equatorial O ions eases, and the Ti atoms reach an equilibrium position with a lower displacement along the *z*-direction. If the strain is applied only in the axial direction, then the behavior is the opposite. A negative strain reduces the distance between the Ti atom and the apical O ion, and the increased repulsion between these two ions compels the Ti atom to displace less from the centrosymmetric position. When the applied strain is positive, the displacement of the apical O atom allows for a larger displacement of the Ti atom away from the centrosymmetric position. In the case of volumetric strain, where the strain is applied in both the equatorial and axial directions, compression of the structure for negative strains implies less available space for the displacement of the Ti atom, while expansion under positive strains enables larger displacements of the Ti atom.

The trends observed at the well depth can be explained in a similar manner. Under a positive axial strain (expansion), there is more space for the Ti atom to separate from the axially polarized O anions. Simultaneously, the apical O anion is positioned further away, reducing the repulsion between the Ti-4*p*_*x*,*y*_ electron and the electronic clouds of the O anions, thereby increasing the well depth. Conversely, when an axial compression is applied to the system, the repulsion between the Ti-4*p*_*x*,*y*_ electron and the O anions increases and the well depth decreases. An equatorial compression destabilizes the centrosymmetric position of the Ti atom in the excited state relative to the noncentrosymmetric position, as the repulsion between the Ti-4*p*_*x*,*y*_ electron and the equatorial O anions increases with the compression. Consequently, the well depth increases with equatorial compression and decreases with expansion. The volumetric strain exhibits a behavior that is intermediate between the observed axial and equatorial trends, as it can be viewed as a combination of both strains.

### Influence of Defects

Extrinsic defects can also substantially modify the electronic properties of STO and induce a ferroelectric phase transition.^26^ However, native defects, such as oxygen vacancies, are also crucial to understanding its behavior. Oxygen vacancy concentration and the conductivity of STO strongly depend on temperature and oxygen pressure, strain, or even thin film thickness.^71–74^ The embedded cluster model is too limited to include an oxygen vacancy, so a 3 × 3 × 3 periodic model has been built to explore the influence of defects on the electronic structure of STO and its photoexcitation. Additionally, comparing the description of the STO electronic structure using both the embedded cluster and periodic models is crucial to ensure the embedded cluster properly captures the crystal field effects. To that end, the band gaps obtained with each approach have been compared. However, this comparison requires careful consideration, as the band gaps obtained are not directly equivalent.^75^ Figure 1a reports the STO optical band gap (3.28 eV) from the combined TD-DFT and cluster model approach. In contrast, most periodic DFT packages calculate the Kohn–Sham band gaps or the fundamental band gaps if quasiparticle energies are used (for instance, using Green’s functions). The inclusion of hybrid functionals with HF exchange partially corrects the underestimation of the Kohn–Sham band gaps, yielding values closer to those of the fundamental band gap. Additionally, optical band gaps are typically slightly smaller than fundamental band gaps, with the difference corresponding to the lowest exciton binding energy.^76^ The indirect HSE band gap of 3.20 eV obtained for the periodic model using plane waves is in good agreement with previous reports^77^ and the values obtained for the embedded cluster model, considering the caveats discussed previously.

The supercell with one oxygen vacancy was optimized using the PBE+U functional, and single-point calculations were used to predict the density of states using PBE+U and HSE06 functionals (Figure 3). Despite the PBE+U band gap underestimation, both functionals present the same qualitative picture, including Ti-filled mid-gap states that reduce the bandgap. The bandgap obtained between the O-2*p* and Ti-3*d* bands is 2.56 and 2.79 eV using PBE+U and HSE functionals, respectively. These values are slightly lower than the ones obtained for pristine STO primitive cells. The gap between the Ti mid gap states and the Ti-3*d* band is smaller than 0.2 eV for the HSE functional and 0.7 eV for the GGA+U functional. The Ti-filled states which represent Ti polarons^78,79^ are located few hundreds of meV below the conduction band which is in agreement with previous reports.^80,81^ The presence of these polarons is unlikely to drastically alter the description of the PE curves for the excited states outlined in the previous section due to their low concentration. However, the existence of electrons that can be excited with energies substantially lower than those in the O-2p band is noteworthy.

**Figure 3:**
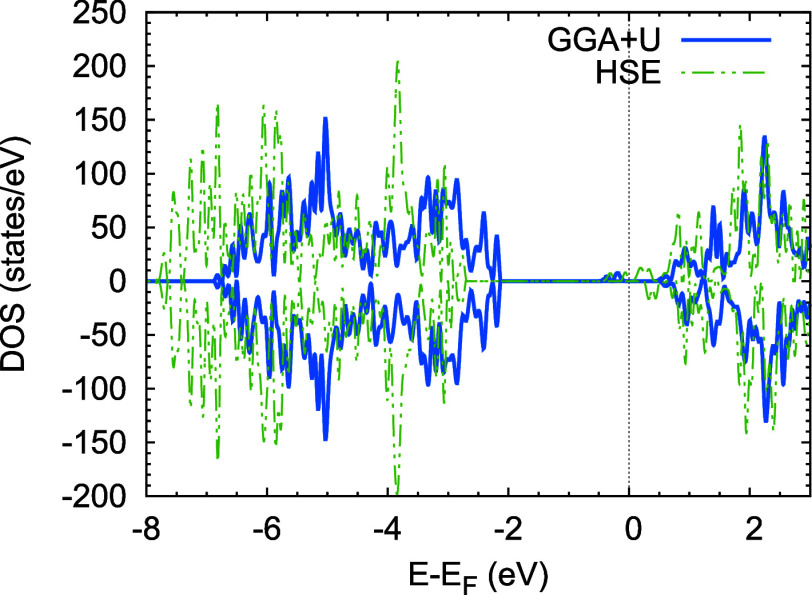
Density of states, DOS, for the STO 3 × 3 × 3 supercell with one oxygen vacancy using GGA+U (blue) and HSE06 (green) functionals.

The transition from a centrosymmetric to a noncentrosymmetric Ti site is one of the necessary conditions for obtaining a ferroelectric state. However, ferroelectricity would be observed only if there is a preferential or net direction for this distortion. The enhancement of the dielectric constant under UV irradiation in the presence of an electric field cannot be considered a ferroelectric effect but rather a dielectric one. The key question is whether it is possible to align the polarons obtained through UV photoexcitation in a preferential direction without an applied electric field in order to induce a ferroelectric instability. Previous examples demonstrate that the perturbations driving the ferroelectric behavior of STO, such as the stress produced by an AFM tip, exhibit directionality. While oxygen vacancies as point defects can locally break the symmetry, it remains to be proven that their long-range distribution is nonhomogeneous and can produce preferential directions. Over the last two decades, the distribution of oxygen vacancies in STO has been extensively explored and even purposefully tailored through experimental studies. Most of these works agree that oxygen vacancy concentrations are higher near (first tens of nanometers or even few microns) the surface.^82–85^ Gentils et al. also described the formation of cation-oxygen vacancies which are not homogeneously distributed.^84^ More recently, Lin et al. have reported colossal room-temperature ferroelectric polarizations in SrTiO_3_/SrRuO_3_ superlattices where oxygen vacancies are accumulated at the interface, breaking the spatial inversion symmetry.^86^ STO ferroelectricity has also been connected to the presence of vacancies in thin films^87,88^ and also nanodots.^89^ In order to explore the oxygen vacancy distribution in the STO bulk, we have built a 4 × 4 × 4 periodic model, 320 atoms, with two oxygen vacancies, SrTiO_3–0.03_. All possible vacancy distributions are analyzed, combining the SOD^90^ package and the high-throughput framework, DisorderNML.^91^ The histogram with the relative energies of the structures with different oxygen vacancy distributions is depicted in Figure 4a, finding significant energy differences between the configurations, up to 0.6 eV. These data quantitatively demonstrate that oxygen vacancies are not randomly distributed, but rather there are some distributions, arrangements, or configurations that are more preferred than others. Despite energy differences between configurations, all of them present the formation of polaron-vacancy complexes, Ti^3+^-O_vac_-Ti^3+^ or Ti^3+^-O_vac_, similar to the experimental results reported by Gentils et al.^84^ This behavior has been already reported in other reducible oxides such as CeO_2_.^92^ The radial distribution function, ρ­(*r*), between polarons, Ti^3+^, has been calculated to describe the distribution of these complexes in the solid (Figure 4b). Two main peaks are found at low temperatures, where only the most stable configurations contribute to ρ­(*r*). The first peak around 4.2 Å represents the formation of the Ti^3+^-O_vac_-Ti^3+^ complexes, while the second peak at 5.8 Å corresponds to the distance between two different complexes. The second peak position is approximately half of the size of the supercell, so Ti^3+^-O_vac_-Ti^3+^ complexes tend to be located as separated as possible. In addition to ρ­(*r*), the orientation of the Ti^3+^-O_vac_-Ti^3+^ complexes for the four most stable configurations is depicted in Figure 4c–f. Our analysis revealed that for all the configurations examined, most of the Ti^3+^-O_vac_-Ti^3+^ or Ti^3+^-O_vac_ complexes were aligned in the same direction. This demonstrates that the oxygen vacancies are not only distributed in a nonuniform manner but also exhibit a preferred directionality in the arrangement of the polaron-vacancy complexes.

**Figure 4:**
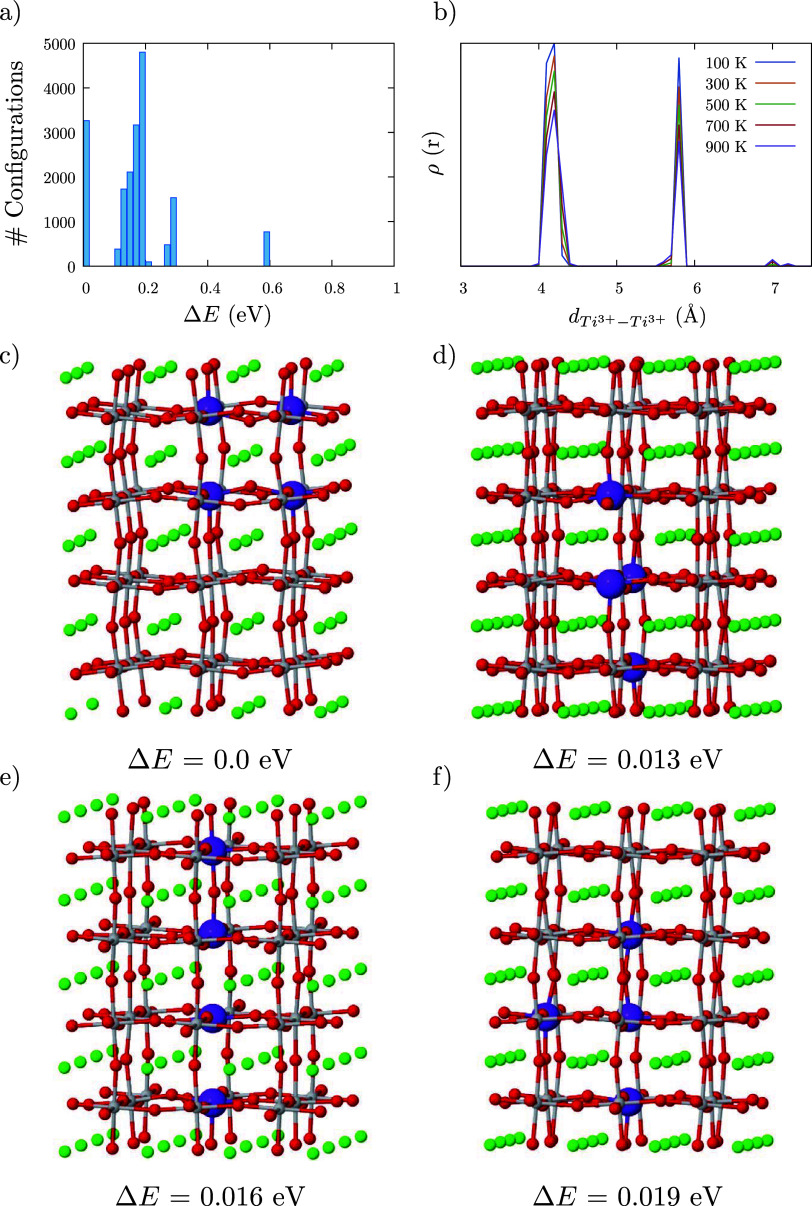
(a) Energy distribution of the different configurations in which 2 oxygen vacancies and 4 Ti^3+^ can be arranged. (b) Average radial distribution function, ρ­(*r*), for the Ti^3+^-Ti^3+^ pairs at different temperatures. (c) Atomistic representation of the ground state configuration. (d–f) Atomistic representation of the other three most stable configurations. Atom colors: red = O, gray = Ti^4+^, purple = Ti^3+^, and green = Sr.

Polarization, **P**, of the 4 most stable configurations was computed as,

$$P=\frac{q_{e}}{\Omega }\sum_{i=1}^{N}Z_{i}\mu_{i}$$ 1

where Ω is the volume of the cell, *N* is the number of atoms, μ is the displacement vector of the *i*th atom relative to the centrosymmetric (stoichiometric) geometry, and *Z*_*i*_ is the Born effective charge tensor of the *i*th atom, calculated using density-functional perturbation theory.^93,94^ The calculated *P* values for the 4 configurations range between 0.36 and 0.1 μCcm^–2^, which are smaller than the values predicted by Son et al. (7 μCcm^–2^).^89^ However, these figures cannot be directly compared due to significant differences in the vacancy concentration, supercell size, and number of vacancies between the two models. Additionally, the choice of exchange-correlation functional plays a crucial role in properly localizing the electrons on the Ti atoms to accurately describe the polarons accurately. To evaluate whether the calculated polarization would be sufficient to align the photoexcited Ti polarons in a common direction due to UV irradiation, we have estimated the equivalent dielectric field that would produce this polarization, considering the calculated dielectric constant (*P* = ϵ_0_ χ_*e*_ *E*). The equivalent electric field is a couple of orders of magnitude greater than the weak electric field applied in the experiments by Takesada et al. (33 V/mm).^45,46^ Therefore, we can conclude that vacancy engineering could be a viable strategy to coordinate the directionality of the photoexcited Ti polarons, moving them from their centrosymmetric positions.

### Conclusions

In this work, we explored the dielectric behavior of STO under UV irradiation and an electric field (Figure 5). Once the electrons are excited to the conduction band (1), the most stable situation is no longer the centrosymmetric solution. The calculated PES shows that the first excited states present a minimum, breaking the centrosymmetric nature of STO (2). The energy difference between the excited state and the GS at this noncentrosymmetric geometry, Δ*E*_3_, is substantially smaller than the main band gap for the centrosymmetric geometry. After relaxation (3), there are two possibilities: (i) phonon modes associated with Ti atom vibration can induce a Ti centrosymmetric position (5) or (ii) a new excitation (4). Considering that electron excitations are faster phenomena than atomic vibrations, intense light sources such as lasers can produce new electron excitations and maintain a nonthermal equilibrium population of the excited states. The minimum energy required for this excitation can be modified under strain depending on how the sample has been experimentally synthesized or processed. To obtain a ferroelectric state under UV irradiation, the electric field should be substituted for another driving force capable of aligning the polarons in a net direction. Based on previous studies linking ferroelectric phase transitions to the presence of oxygen vacancies in various STO thin films and nanostructures, we have explored the oxygen vacancy distribution in bulk STO. Preferential oxygen vacancy distributions have been found, in which Ti^3+^-O_vac_-Ti^3+^ or Ti^3+^-O_vac_ complexes were aligned in the same direction. Small polarization was found in these configurations, which could be used as the driving force to align the off-center photoexcited Ti polarons.

**Figure 5:**
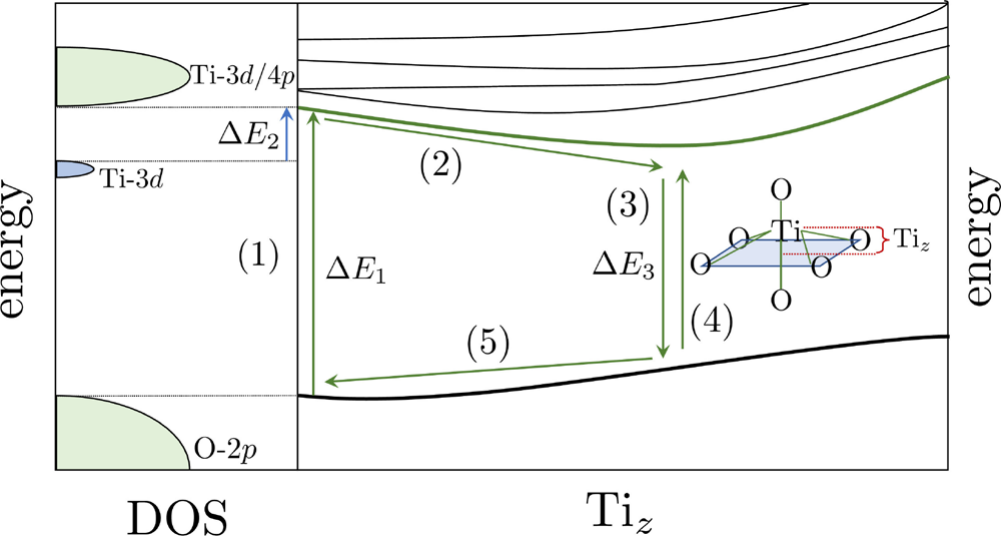
Proposed mechanism to explain the photocurrent observed on STO using a radiation whose energy is below the fundamental band gap.
